# Social Cognition and Executive Functions As Key Factors for Effective Pedagogy in Higher Education

**DOI:** 10.3389/fpsyg.2017.02016

**Published:** 2017-11-21

**Authors:** Rut Correia, Gorka Navarrete

**Affiliations:** ^1^Facultad de Educación, Universidad Diego Portales, Santiago, Chile; ^2^Center for Social and Cognitive Neuroscience, School of Psychology, Universidad Adolfo Ibáñez, Santiago, Chile

**Keywords:** social cognition, executive function, teaching effectiveness, higher education, theory of mind, empathy

## Abstract

Higher education (HE) faces the challenge of responding to an increasing diversity. In this context, more attention is being paid to teachers and teaching skills positively related to students learning. Beyond the knowledges identified as key components of an effective teacher, teachers also need to be capable of unraveling what their students think and believe, and how they accommodate the new information. More importantly, teachers need to be able to adapt their own teaching to their audience’s needs. In learners, social cognition (SC) has been related to a better receptivity to the different teacher-student interactions. Since these interactions are bidirectional, SC could also help to explain teachers’ receptiveness to the information available in feedback situations. However, little is known about how SC is related to teacher development, and therefore teaching effectiveness, in HE. In addition, executive functions (EFs), closely related to SC, could play a key role in the ability to self-regulate their own teaching to better answering their students emerging needs. Although there is wide evidence regarding the association of EFs to performance in high demanding settings, as far as we know, there are no studies exploring the relationship between teachers’ EFs and teaching effectiveness in HE. Establishing a positive association between teaching effectiveness and these socio-cognitive functions could be a promising first step in designing professional development programs that promote HE academics’ ability to understand and care about students thoughts and emotions, to eventually adapt their teaching to their students needs for a better learning.

Higher education (HE) deals with students increasingly diverse in a wide range of variables such as age, gender, race, ethnicity, academic and socioeconomic backgrounds, among others (Smith, 1989; Gurin et al., 2002; Brown, 2004). As a consequence, offering high quality learning opportunities so all the students reach the expected achievements emerges as a great challenge for Universities these days. Since teachers are the ones engaged in a closer and more frequent interaction with students, it seems reasonable to think that whatever strategy Universities implement to deal with this challenge, teachers should be a nuclear part of it Lazerson et al. (2000) and Barrington (2004).

In a context where students are no longer the only ones responsible for their learning, more attention is being paid to teachers and teaching characteristics that are positively related to students learning (Fry et al., 2003; Klassen and Tze, 2014). Some authors suggest that these fundamental characteristics do not differ significantly across different levels of Education (Hutchings and Shulman, 1999; Lazerson et al., 2000). In this sense, Pedagogical Content knowledge, emerging from both Subject matter and Pedagogical knowledge, has been identified as a key component of an effective teacher (Shulman, 1986). Particularly in experienced HE teachers, Pedagogical content knowledge has been assumed, since their pedagogical experience is already framed in their own disciplines (Lazerson et al., 2000). However, it has been suggested that effective teachers also need to be capable of unraveling how their students understand and accommodate the new information, so they can adapt their own teaching to their audience’s particular needs (Darling-Hammond, 1998).

The former proposal for teacher effectiveness (Darling-Hammond, 1998), describes the teaching-learning process as an interaction that in order to be successful needs the information to flow not only from the teacher to the student, but also in the opposite direction (Battro et al., 2013; Mcconville, 2013; Watanabe, 2013). The importance of this bi-directionality could be even greater in a context of growing diversity, where teaching designed for one particular student profile may not be effective in engaging the motivation of all learners and offering them optimal learning opportunities (Guri-Rosenblit et al., 2007). Nonetheless, the importance of this bidirectional interaction for a better student achievement does not seem to have fully permeated the actual practices of HE teachers (Chang et al., 1981; Tettegah and Anderson, 2007), where teaching effectiveness is mostly still assessed through traditional measures that seem non-related to student learning (Uttl et al., 2017), and teacher cognitive and socio-emotional competencies are largely overlooked.

Although most of the scientific evidence supporting the importance of this bidirectional interaction for teaching effectiveness comes from school settings (Lucariello et al., 2016), there are some experiences that highlight the importance of this interaction also in HE. In a qualitative study from the Harvard Graduate School of Education (Rodriguez and Solis, 2013) 23 master teachers were asked about “*What are you focusing your mind on throughout the process of teaching?*” (Rodriguez and Solis, 2013, p. 161). Participants in the study varied from Pre-K teachers to graduate-level professors, and were selected because they had previously been recognized for their teaching effectiveness. The authors conclude that teachers’ responses reveal that the awareness of the learner–teacher interaction is fundamental for a successful learning. In this sense, they identify three main awareness dimensions in teachers’ responses critical for a successful learning:

(1) *Connection:* described as the close relationship with the student, the need of creating a true understanding of the other, the importance of sharing feelings.
(2) *Collaboration:* reveals the former interaction as an active process for both, the teacher and the student, who work together toward a common goal.
(3) *Mutual effects:* is the awareness of some sort of Banduras’ Reciprocal Determinism (Bandura, 1978, 1989). Teachers realize when they adapt their teaching to respond to students feedback, students respond changing their approach to learning.

Similarly, Bain (2004), after his analysis on the practices and characteristics of the “What the Best College Teachers,” highlights that *the best teachers* share a relationship of trust with their students and value the interaction with them. Although systematized information about non-effective teachers is lacking, this qualitative evidence suggests that master teachers seem to have developed a high level of theory of mind (ToM) and Empathy. Both ToM and Empathy are core components of what is known as social cognition (SC), that is, the set of cognitive processes that enable us to interact effectively and safely with other people (Adolphs, 2009).

Theory of mind, defined as the ability to infer our own and others’ mental states that can be used to predict the behavior of others (Premack and Woodruff, 1978), has already been presented as a critical ability that allows teachers to engage in a successful interaction with their students (Strauss and Ziv, 2012; Mcconville, 2013; Rodriguez, 2013). Research on the relationship between teachers’ ToM and teaching effectiveness has traditionally been approached by studying teachers’ beliefs about learning (Strauss and Shilony, 1994; Strauss et al., 1998). These studies focus on teachers identification of key concepts for students learning and provide rich information about the learning theories that teachers implicitly or explicitly share. However, they are not informative about teachers’ ToM, that is, they tell little about teachers’ ability to read their students thoughts, needs or intentions when interacting with them. Despite a growing consensus about its importance, as far as we know, no studies have been published so far aiming to identify the cognitive processes that allow teachers to understand their students’ thoughts, intentions and needs. More specifically, no studies have been published that assess teachers ToM and explore the relationship that could exist between teachers’ ToM and their performance or their students learning.

Together with ToM, Empathy is the other main SC component. It is known as the ability to not only recognize or identify others’ feelings, but also to experience these emotions by adopting their perspective and responding with sensitivity and concern to their suffering or needs (Batson, 2009). In the educational context, it has been emphasized the importance of knowing how to communicate that we have indeed understood the other’s feelings and our will to help (Feshbach and Feshbach, 2009). Some authors have suggested that empathic teachers model and facilitate their students learning and empathic development (Chang, 2003; Cooper, 2004). It has also been argued that empathic teachers promote their students positive attachment to them and to schools (Carkhuff and Berenson, 1967). To date most studies on empathy in educational contexts have approached empathy from the Rogers therapeutic perspective (Feshbach and Feshbach, 2009). This perspective posits that empathy in educational contexts works as in therapeutic settings, that is, the more communicative and understanding the teacher is with their students, the greater the bond between students and teachers becomes, at the same time promoting students bond with school (Rogers, 1969). In this sense, the meta-analysis performed by Cornelius-White (2007) including studies from 1942 to 2004, confirms the positive relationship between “positive personal characteristics of the teacher,” such as empathy, and positive students behavior. In addition, this relationship seems to be independent from the teacher previous pedagogical experience. Nevertheless, this comprehensive meta-analysis also reveals some limitations of the current knowledge and establishes challenges for future research such as: (a) the need for more objective measures of empathy, moving away from self-reported measures (Stueber, 2017), (b) the need to further explore the relationship between teachers empathy and teachers performance, and (c) the need for this relationship to be studied specifically in HE, where it has received much less attention.

As opposed to teachers’ SC, learners’ SC has been widely related to performance and academic achievement. A recent review points out that SC in children is not only positively related to specific academic skills such as reading and writing but also predicts the development of metacognitive skills throughout childhood (Wellman, 2016). Although this review makes no mention about how teachers’ SC could impact teaching-learning interaction, it highlights two arguments that are central for this *perspective* article: (a) children with greater SC are more receptive to information available in feedback instances; and (b) SC processes are trainable.

Regarding the first argument, little discussion exists today on the interactive nature of teaching-learning processes. Thus, when recognizing the importance of the learning mind and brain, researchers should not forget about the other mind and brain involved in the interaction: *the teaching brain* (Rodriguez, 2013). In this sense, Wellman argument invites us to think that teachers with greater SC should make the most of the interactive instances with their students, and would be more receptive to their students needs, thoughts, etc. The second argument presented by Wellman points to the importance of studying the relationship between teachers’ SC and their performance or effectiveness. If this relationship proves to be positive, emphasizing the development of SC abilities could contribute to the birth of a new way to train HE teachers. In the light of some promising evidence of adults’ SC being amenable to intervention (Horan et al., 2008; Santiesteban et al., 2012; Bishop-Fitzpatrick et al., 2013) and a few attempts aiming to intervene in teachers development of social skills (Barton-Arwood et al., 2005; Talvio et al., 2016; Jennings et al., 2017), helping teachers to further develop their SC could become an evidence-based strategy to enhance teachers cognitive development and therefore effectiveness in HE, but more evidence linking these interventions to a positive impact in students learning is needed.

In addition to all the above, being aware of what their students are understanding and learning should be a very useful tool for teachers to timely self-regulate their own teaching. Moreover, effective teachers also need to monitor, assess and reflect on their own teaching performance, as well as having the flexibility to implement the necessary changes to improve it Darling-Hammond (1998). Therefore, effective teachers first need to integrate the information coming from their students with the information from their own behavior and, then, make the necessary adjustments to offer an inclusive learning experience. In cognitive terms, an effective teaching would demand a good executive functioning. Although different conceptualizations of executive functioning have been proposed depending on the specific processes being emphasized, there is a general consensus in defining executive functions (EFs) as a set of processes in charge of planning, monitoring and regulating behavior in relation to an established goal (Stuss and Alexander, 2000; Alvarez and Emory, 2006; Flores and Ostrosky, 2012; Lezak et al., 2014). EFs have been extensively linked to academic success (Meltzer, 2007; Best et al., 2011; Samuels et al., 2016), as well as to professional performance in some highly demanding contexts (Stavrakaki et al., 2012; Vestberg et al., 2012). As far as we know, there are no studies exploring the relationship between EFs and effective teaching, but a very recent study on teachers’ temperament found that the *conscientiousness* personality trait is positively related to some external measures of teaching effectiveness in school *first-year* teachers (Bastian et al., 2017). A C*onscientiousness personality* is related to an organized and planful, achievement-oriented worker (Barrick and Mount, 1991), suggesting a high similarity with the set of cognitive functions identified as EF. Therefore, although prior evidence suggests that EF might be an important factor behind teacher effectiveness, we have not found studies exploring this relationship in HE.

Literature from cognitive and social neuroscience shows a close relationship between EFs and SC in both children (Sabbagh et al., 2006) and adults (Saxe et al., 2006). The nature of this relationship has not been yet clarified, and while some studies suggest that EFs underlie SC, particularly ToM (Baez et al., 2014), others argue this relationship is based on the overlap of some neuroanatomic circuits (Saxe et al., 2006). In any case, a review on the relationship between EFs and SC in patients with acquired neurological pathology defends CS and FE as distinct cognitive functions (Aboulafia-Brakha et al., 2011), although a positive relationship between them has been consistently reported. These authors also emphasize the need to further explore this relationship in different contexts and populations since there is no agreement about which processes are shared by both functions.

At present the influence of SC in learning has received considerable attention, mainly from the study of these abilities in learners. In comparison, the study of teachers’ SC and how these abilities are related to teachers development and teaching effectiveness has been widely ignored. Although this relationship has been theoretically defended in previous work, empirical approaches focusing in HE are scarce and show some important methodological flaws. In addition, the consistent association found between EFs and SC makes critical to include teachers’ EFs when exploring the association between teachers’ SC and effective teaching in HE. On one hand, it would help to clarify whether teachers’ EFs is to some extent related to teacher effectiveness in HE. And on the other hand, it would be necessary to explore if teachers’ EFs have a mediating role on the relationship between teachers SC and their teaching effectiveness.

In this context, research on teachers’ EF and SC in relation to teachers effectiveness in HE seems auspicious, but further empirical efforts need to be made. In a situation of growing diversity, HE teachers are in need of an upgraded toolkit of teaching strategies and skills to offer appropriate learning opportunities to all their students. Although establishing a positive association between teachers’ SC and EFs and their teaching effectiveness is not enough to infer a causal relationship, it could be a helpful first step. If this relationship can be established, specific programs could be implemented in HE in order to assess the impact they could have in teachers development and teaching effectiveness. Eventually, professional development programs offered by Universities in order to improve their academics teaching skills, could highly benefit from implementing changes based on this expected evidence.

## Author Contributions

Both authors listed RC and GN have made substantial, direct and intellectual contribution to the work, and approved it for publication.

## Conflict of Interest Statement

The authors declare that the research was conducted in the absence of any commercial or financial relationships that could be construed as a potential conflict of interest.
